# Moral entrepreneurship, the power‐knowledge nexus, and the Cochrane “crisis”

**DOI:** 10.1111/jep.13124

**Published:** 2019-03-18

**Authors:** Trisha Greenhalgh, Mustafa F. Ozbilgin, Barbara Prainsack, Sara Shaw

**Affiliations:** ^1^ Nuffield Department of Primary Care Health Sciences University of Oxford Oxford UK; ^2^ Brunel Business School Brunel University London London UK; ^3^ Department of Political Science University of Vienna Wien Austria; ^4^ Department of Global Health and Social Medicine King's College London London UK

**Keywords:** evidence‐based medicine, philosophy of medicine, systematic reviews

## Abstract

**Background:**

In 2018, a so‐called crisis developed in the international network of systematic reviewers known as Cochrane. It was widely depicted in terms of two competing narratives—“bad behaviour” by one member of Cochrane's Governing Board and scientific and moral decline within Cochrane.

**Objective:**

Our goal was to distil insights on the structural issues underpinning the crisis, without taking a definitive position on the accuracy of either narrative.

**Approach and dataset:**

In this paper, we draw on (among other theories) Becker's notion of moral entrepreneurship and Foucault's conceptualisation of power to analyse the claims and counterclaims made by different parties. Our dataset consisted of publicly available materials (blogs, journal articles, newspaper articles) to end 2018, notably those relating to the expulsion of one Governing Board member.

**Main findings:**

Both narratives include strong moral claims about the science of systematic review and the governance of scientific organizations. The expelled individual and his supporters defined good systematic reviews in terms of a particular kind of methodological rigour and elimination of bias, and good governance largely in terms of measures to achieve independence from industry influence. Most of Cochrane's Governing Board and their sympathizers evaluated systematic reviews according to a broader range of criteria, incorporating factors such as attention to relationships among reviewers and reflexivity and dialogue around scientific and other judgements. They viewed governance partly in terms of accountability to an external advisory group. Power‐knowledge alignments in Cochrane have emerged from, and contributed to, a particular system of meaning which is now undergoing evolution and challenge.

**Conclusion:**

Polarizing Cochrane's “crisis” into two narratives, only one of which is true, is less fruitful than viewing it in terms of a *duality* consisting of tensions between the two positions, each of which has some validity. Having framed the conflict as primarily philosophical and political rather than methodological and procedural, we suggest how Cochrane and its supporters and critics might harness their tensions productively.

## 1 BACKGROUND: TWO NARRATIVES OF A CRISIS

In the 26 years since its inception, the international network of systematic reviewers known as Cochrane (previously, the Cochrane Collaboration) has grown from a tiny group of academics run on collegiality and small donations to an extensive transnational bureaucracy with numerous subcommittees, a thick tome of standard operating procedures, and a multimillion dollar annual turnover along with an address book of philanthropists (including some with links to industry) on whose contributions its work depends.^1^

At the time of writing, at least two competing narratives are circulating of a crisis (or, perhaps, a perceived crisis): a narrative of “bad behaviour” by one individual and a counternarrative of moral cowardice and scientific decline within Cochrane itself.^2^ The story is still unfolding; both sides have consulted lawyers, and some key evidence is not in the public domain. Nevertheless, it is time to take stock and to try to understand the wider significance of the controversy and the structural issues that lie at its root. In preparing this paper, we have drawn on a dataset of material in the public domain that referred to the Cochrane crisis, as well as selected materials cited by those sources, including blogs, newspaper articles, books, academic papers, and official statements issued by the Cochrane Governing Board.

This paper begins from the philosophical position that reality is multifaceted and multilayered. This means that more than one perspective—and hence more than one version of the truth—on this crisis may exist. We do not, however, accept the relativist view that every version holds equal weight. We also take the position that whilst reasonable people may disagree on what is good science or good governance, such disagreements can be usefully illuminated through the use of theory. Below, we first present the crisis as conventionally depicted—as two different, antagonistic and mutually exclusive narratives—but we will go on to show how they can be usefully combined using higher‐order theory.

The official narrative from Cochrane's Governing Board is that Professor Peter Gøtzsche, a founder member of the Cochrane Collaboration and Head of the Nordic Cochrane Centre, was removed from the Board in September 2018 for *“a long‐term pattern of behaviour that we say is totally, and utterly, at variance with the principles and governance of the Cochrane Collaboration,”* and which interfered with *“the right [of Cochrane Collaboration staff and members] to do their work without harassment and personal attacks.”* 3 Gøtzsche was also accused of scientific bias, allegedly misusing Cochrane letterhead (and hence the valued Cochrane brand) to express personal opinions, and (implicitly) of bringing Cochrane into disrepute.^2^, ^4^

This narrative depicts Gøtzsche as an intellectual maverick who took an extreme position on key scientific questions including mammography screening programmes, which he believed should be curtailed,^6^ antidepressant drugs, which he believed were usually unnecessary,^7^ and vaccination, whose benefit‐harm ratio he believed had been overestimated.^7^, ^8^ Gøtzsche is also criticized for having allegedly ignored or dismissed evidence that did not support his chosen position, put pressure on the Danish government to change policy in line with his views, made a personal profit from books and paid lectures that presented a distorted version of the truth, and reacted in a hostile way towards both academic and financial oversight of his work.^5^, ^7^

The alternative narrative is that Gøtzsche, a heroic defender of high scientific (and especially methodological) standards, has been unfairly punished in the context of a longstanding crisis of governance in Cochrane. In a recent case in point, Gøtzsche and colleagues published a detailed critique of a newly published Cochrane review on human papilloma virus (HPV) vaccine in cancer prevention,^8^ claiming that the authors had failed to identify numerous studies and misapplied the Cochrane risk‐of‐bias tools, resulting in a review that was itself biassed.^9^ This prompted an editorial in the journal *BMJ Evidence‐Based Medicine* defending Gøtzsche's team,^10^ although at least one leading scholar considered their analysis flawed^7^ and Cochrane's Editor‐in‐Chief ruled that what had been described as “omissions” were actually the result of defensible judgements that took account of clinical, scientific, and policy realities.^11^

The “moral and scientific decline” narrative depicts Cochrane's central executive as having condoned sloppy standards, micromanaged national centres, suppressed scientific debate among its members, and progressively sold out to commercial, policy, and other vested interests.^12^, ^13^, ^14^, ^15^ Gøtzsche has, so this narrative goes, meticulously exposed the widespread conflicts of interest—especially paid advisory roles to industry—among Cochrane members and remained steadfast in the face of oppressive tactics from its executive.^16^

Given that the Cochrane name has long been associated with scientific rigour, neutrality, and a commitment to the public good, these depictions of the Governing Board's scientific and moral regression are dramatic and shocking. Indeed, there is a David‐and‐Goliath quality about the story of one clear‐sighted scientist single‐handedly taking on a once‐noble and still‐mighty organization (*“the boy who sees the emperor has no clothes and says so”*).^17^ Importantly, this narrative has been progressed by a network of internationally renowned academics, who penned an open letter to the Danish Minister of Health.^18^ In a social media recruitment effort that had hints of what sociologists have called moral panic,^19^ the letter was signed by almost 9000 people.^18^ Many of Gøtzsche's supporters are longstanding members of Cochrane; four were on its Governing Board and resigned after being outvoted on the decision to oust him.^20^, ^21^

In the next two sections, we will analyse these two narratives using different theoretical lenses and develop an argument that the various competing moral claims reflect a *duality* of two seemingly incommensurable positions that exist in tension with one another. We will also argue that this particular crisis, whilst appearing to centre on a unique case about a single individual, is symptomatic of a wider unease in contemporary scientific practice—which on the one hand operates with the main goal of truth seeking and on the other cannot function without its own structures of power and ranking that sometimes contradict its main goal.

## 2 MORAL ENTREPRENEURSHIP

In 1963, in the context of his work on how society deals with deviants or outsiders, sociologist Howard Becker described a type of person that he called the moral entrepreneur, which he believed involved two kinds of individual.^22^ The crusading reformer or “rule maker,” suggested Becker, *“… is interested in the content of rules. The existing rules do not satisfy him [sic] because there is some evil which profoundly disturbs him. He feels that nothing can be right in the world until rules are made to correct it.”* (page 147).

Becker observed that whilst such reformers typically come across as fervent and self‐righteous, they are often driven by humanitarian motives and can potentially achieve a great deal of good. The second type of moral entrepreneur, he proposed, is the “rule enforcer,” who may or may not hold strong moral views about the rules but views their job as applying the rules in practice. As Becker puts it, *“Just as radical political movements turn into organised political parties and lusty evangelical sects become staid religious denominations, the final outcome of a moral crusade is a police force”* (page 48).^22^

The term “police force” is used metaphorically—and probably ironically—by Becker to depict people whose job is to enforce whatever rules currently prevail—a task that Becker viewed as pragmatic and context dependent. As rules change, rule enforcers shift their activity accordingly—usually without experiencing moral dissonance. According to Becker, as well as internalizing the rules, rule enforcers also tend to develop an unwritten hierarchy of the relative importance of different rules (since they may conflict in practice) and a set of subjective heuristics which guide the application of rules in the real world.

Through the lens of moral entrepreneurship, Gøtzsche and a small group of his colleagues could be viewed as knowledgeable rule makers and other Cochrane members, including some senior figures and Governing Board members, as imperfectly performing rule enforcers. The rule makers see their mission as morally driven (hence, virtuous) and uncompromising. As Gøtzsche wrote in a blog for the *British Medical Journal* in November 2018^23^:

“Eighteen months ago, I was elected to Cochrane's Governing Board with the most votes of the 11 candidates. My aim was to stop the rot—what I saw as a moral slide—and I challenged the leadership on core issues and on the way it was managing the charity. […] I tried to block the CEO from micromanaging centres, so researchers could be free to operate autonomously with their own funding, but failed.

I wrote a policy a year ago that would prevent Cochrane authors from having a commercial interest in the interventions they were assessing. The Cochrane leadership stalled.”

Notwithstanding Gøtzsche's claim to democratic legitimacy (on the grounds of having the “most votes”) in the quote above, it is sometimes hard to decide whether the actions of a group of moral entrepreneurs are virtuous or vicious. Such decisions require us to make a judgement that takes account of the particular social context (what Bourdieu has called the “field of relations”^24^) in which the moral entrepreneurship takes place and gains moral meaning. The rule enforcers, as fellow moral entrepreneurs, adopt the rule maker's espoused moral principles into systems, structures, and processes. As ideals of the moral entrepreneur become entrenched in the institutional setting, the consequences of moral entrepreneurship may defeat scrutiny in a process which Bourdieu calls *toxic illusio*—the allure of a game that draws participants in whilst at the same time preventing them from developing a healthy distance and critical perspective about the consequences of the game for its varied stakeholders and participants.^25^ As Hilgers and Mangez have put it:

“The greater its autonomy, the more the field is produced by and produces agents who master and possess an area of specific competence. The more it functions in accordance with the interests inherent in the type of activity that characterizes it, ‘the greater the separation from the laity’ (Bourdieu 2000: 58^24^) and the more specific become the capital, the competences and the ‘sense of the game’. This closure is an index of the autonomy of the field. It is for the politician to speak of politics, for writers to speak of literature, and so on. As the field closes in on itself, the practical mastery of the specific heritage of its history, objectified and celebrated in past works by the guardians of legitimate knowledge, is also autonomized and increasingly constitutes a minimum entry tariff that every new entrant must pay. The autonomization of a domain of activity generates the doxa, an illusio that forms the prereflexive belief of the agents of the field, i.e. a set of pre‐suppositions that implies adherence to a domain of activity and implicitly defines the conditions of membership.”^26^ (page 7)

The case being made by Gøtzsche and those who might be viewed as his fellow moral entrepreneurs appears to rest on three key arguments. First, that systematic review is essentially a technical task rather than a broader analytical and critical process, and that its success depends largely on rigorous application of standardized tools and approaches. Second, that it is both desirable and possible to remove all conflicts of interest in Cochrane (and in science more generally). Third, there should be no restrictions placed on “academic freedom.” We take these arguments in turn.

Gøtzsche has argued—controversially—that content experts may not be required on systematic review teams since assessing methodological quality is an almost exclusively technical task; hence, rigorous critical appraisal carried out by methodological experts will, to a large extent, reduce bias and thereby help reveal the truth.^27^ This position reflects what is known as the central limit theorem—that a single version of the truth exists and that it is the role of science to reveal it.^28^ Experiments, to the extent that they eliminate bias, can get us closer and closer to that unitary truth. With this perspective, disagreements between reviewers tend to be attributed to methodological errors rather than to differences in meaning and interpretation. Taken to extreme, such a position would hold that there are only good or bad reviews and good or bad reviewers^28^ and that failure to confront the badness reflects “rot” in Cochrane's processes.^23^ This vision of science assumes that rigour has no moral or value base and that systematic review (and reviewers) can be free from value judgements. This view (which has been challenged by others^29^, ^30^) further implies that explanations should be so robust as to be undisputable and that a key route to robustness may be to reject disagreement.

An alternative perspective holds that there are always value judgements involved in framing a scientific question and weighing the evidence that addresses it; hence, every statement about the world inevitably also includes interpretation.^31^ Scientific facts, therefore, are not self‐interpreting, and as such they are theory and value laden.^31^, ^32^, ^33^ From this perspective, meticulous application of the Cochrane Handbook by skilled reviewers is likely to generate new kinds of disagreements rather than a single, uncontested truth.^31^ Even when there are agreed criteria for including or excluding a study or for assigning a particular score to the methods (for example, using a risk‐of‐bias scoring tool), multiple value judgments need to be made.^7^ What if one trial used a slightly different version of a vaccine, or a different test for a primary end‐point, than the one named in the protocol? Because of the need for judgement on such questions, two systematic review teams can produce different findings even when both teams are equally competent and rigorous, and they use identical checklists and statistical methods.^34^

The second moral claim made by Gøtzsche and his supporters relates to conflicts of interest, especially around the pharmaceutical industry. Such conflicts in clinical trials and meta‐analyses are typically associated with skewed findings in favour of the sponsor's product.^35^, ^36^, ^37^, ^38^ A counsel of perfection would exclude all external sponsorship, direct or indirect, from all Cochrane work, and ban reviewers with any hint of industry ties. But systematic reviews are expensive, especially as methods become more labour intensive (eg, involving reanalysis of individual patient data). Furthermore, only a tiny fraction of senior researchers can boast no industry connections at all, and any large international collaborative endeavour needs infrastructure funding.

Whilst we do not, for the reasons explained above, believe that fully disinterested, dispassionate, value‐free science is ever possible, we do believe that instruments such as transparency about one's own assumptions and interests can help to produce better science. We thus fully agree with an approach that requires reviewers to be explicit about their own stakes, and for their impartiality to be challenged if they have concealed these or have an unduly narrow understanding of what could constitute a conflict of interest. In the same vein, introducing appeal mechanisms may render scientific inquiry more accountable to public.

Cochrane has a conflict of interest policy, overseen by a funding arbitration panel, which explicitly and categorically bans *“conflicts of interest associated with commercial sponsorship”* (although not necessarily all forms of industry support) for funding specific reviews; it also bans involvement or interference in reviews by anyone with a vested interest.^39^ But the wording of the policy includes a contestable grey zone. For example, researchers who have undertaken commercially sponsored trials in the past must declare this but are not barred from being reviewers. The moral entrepreneurs would like this policy to go much further and are unsympathetic to pragmatic arguments about funding. The author of a recent book on the Cochrane Collaboration reports on an interview with Gøtzsche:

“Funding challenges remain, however. As Peter Gøtzsche explains, ‘The workload of Cochrane review groups increases exponentially. It is untenable, and we just need more funding for the Cochrane review groups.’ But should that money come from industry? ‘No,’ he says, ‘It should come from governments.’” (page 126)^1^

The moral entrepreneurs' third argument centres on freedom of speech—a principle dear to the hearts of many academics. Surely scientists should be allowed to speak the truth. Yet a critical question is freedom of speech *for whom*? The example above about HPV vaccine illustrates that contrary to some claims, Gøtzsche and his colleagues' views were not censored. They were disseminated widely as scientific articles,^9^, ^10^, ^40^ blogs,^41^ and online videos,^42^, ^43^ as were the perspectives of reviewers and editors who disagreed with them.^7^, ^11^

The Cochrane Governing Board were adamant that they had no objection to Gøtzsche publishing his views as an independent scientist (although they had asked him to respect confidentiality of documents exchanged during ongoing disputes with the Board). What they objected to was him conflating his personal scientific views with the position of the Nordic Cochrane Centre (hence, in effect, trading on the Cochrane brand).^3^, ^4^ Gøtzsche's supporters responded that he was fully entitled to speak for the Nordic Cochrane Centre because he was such an exceptionally good scientist. In other words, they framed him as the ultimate rule maker and his national centre as a rule enforcer:

“The CEO [of the Cochrane Collaboration] … believes Gøtzsche is bad for the ‘brand’. Not being a scientist, he has no recognition that the work Gøtzsche does IS the brand. The ideal of science is not sitting by idly while science is perverted by moneyed interests. The ideal is someone who thoroughly vets the science being produced and is willing to challenge corruption at great personal risk.” 44

In contrast, the Governing Board's perspective was that what Gøtzsche viewed as “freedom of speech” was actually a longstanding pattern of confrontational behaviour which some of his fellow board members and reviewers experienced as “harassment” and “personal attacks.”^3^ Gøtzsche's supporters strongly resisted this position:

“… . ‘personal behaviour’ is being used to avoid a serious debate on the future strategy and policies of the organization. Of course, there are all kinds of people with different characters and different temperaments as in any large organization. Yes, there have been some passionate and sometimes overly heated discussions concerning important policy issues of Cochrane in which both the Cochrane leadership, including its CEO, and Peter Gotzsche have been involved. But this crisis is not about style but substance.” 14

The moral rules articulated by Gøtzsche and his colleagues—methods and tools over‐riding interpretation and judgement; a somewhat monastic approach to conflicts of interest; academic freedom defined as declaring one's views however and whenever one wishes—could be interpreted by some as fundamentalist. Under what might be called the spell of a Bourdieusian illusio, underpinned by the halo created by the supposed success and growth of the evidence‐based medicine movement over the years, these moral entrepreneurs may occasionally fall short on critical self‐reflection.

In sum, Gøtzsche and his fellow moral entrepreneurs—both the handful of leaders and the thousands of followers who put their names to the protest letter—have depicted as intellectual rigour (a virtue) what others have interpreted as intellectual rigidity (a vice). Their dogged insistence on this framing arguably reflects moral and philosophical immaturity, which lead to usurpation of voice and power from the less powerful resistance for the sake of academic consistency and rigour. Relevant here is Thomas Kuhn's notion of scientific paradigms, which become obsolete over time as empirical evidence emerges that does not fit the existing normal science.^44^ Relevant too is Paul Feyerabend's work, who argued in *Against Method* that theoretical pluralism is more likely to encourage progress than its law‐and‐order alternatives.^45^ He also argued that, although modern science has given us theories of great beauty and sophistication, they are problematic to the extent that they conceal underlying troubles.

## 3 FOUCAULT AND THE POWER‐KNOWLEDGE NEXUS

Michael Foucault, who depicted power and knowledge as intertwined and interdependent, emphasized the importance of discourse in generating and maintaining power‐knowledge alignments.^45^ Foucault defined discourse as a system of language use and other meaning‐making practices (eg, behaviour, dress, and customary practices) that form ways of talking about and enacting social reality. Language does not merely describe the world; it creates and shapes it (for example by making some things appear correct or reasonable or worth studying and other things incorrect, absurd or not worth studying). Indeed, Foucault proposed, it is impossible for language to serve as a neutral conduit for the truth. He recommended a close and critical reading of language to surface the (often competing) discourses within which we are all, largely unconsciously, immersed.

Foucault's notion that objects, subjectivities, and events come into existence as meaningful entities through discourse helps explain why the “bad behaviour” and “scientific and moral decline” narratives are structured almost as mirror images of each other. In the former account, the Cochrane Collaboration is virtuous and well governed whilst Gøtzsche is a flawed character who resists the Collaboration's governance mechanisms and cannot effectively govern his own Centre. In the latter account, it is Gøtzsche who is virtuous and committed to good governance whilst Cochrane (and, to a lesser extent, the Rigshospitalet too) is a flawed organization that has strayed from the rules, allowed governance to slip, and begun to persecute the story's hero.

In both these narratives, the rhetoric is strong and morally loaded—and it is accompanied by morally symbolic actions. The Cochrane Governing Board chose to use the parent‐child language of “bad behaviour” to describe Gøtzsche's actions and mobilized both internal governance processes and external lawyers to support their actions against him. In return, Gøtzsche has accused Cochrane's Governing Board of discrimination and his bosses at the Rigshospitalet of *“scientific judicial murder”* 46; he has declared that *“I am known for high quality research, integrity and incorruptibility”* 46 and opened a GoFundMe page seeking donations for the defence of *“scientific freedom, honesty and integrity.”* 47 One supporter has framed the Board's actions against Gøtzsche as “*The crucifixion of Brother Peter*.”^17^ As this paper went to press, Gøtzsche published a book entitled *“Death of a whistleblower and Cochrane's moral collapse.”* 48

A significant difference in the two narratives is their contrasting use of the term “governance.” For Gøtzsche and his supporters, governance seems to equate to strict scientific and technical standards and an adversarial and exclusionary relationship with industry. The Cochrane Governing Board, in contrast, define good governance in much broader and more participatory terms. A strategic review in 2009 acknowledged Cochrane's rapid growth, increasing geographical reach, and close links to national and international policy.^49^ That review set broad goals including (in addition to, and with the ultimate purpose of, producing high‐quality systematic reviews) developing cross‐sector partnerships, developing and periodically appraising the performance of its leaders, and overseeing the work of Cochrane entities around the world *“to ensure efficient alignment with the purposes of the Collaboration”* (page iii).^49^ Citing existing Cochrane policy, the review recommended that chairs of Cochrane entities are expected *“… to have leadership skills and to be fully consultative, to have vision, to be adept at dealing with people, to be able to solve problems and resolve conflicts effectively, to communicate well, and to have the self‐confidence to represent The Cochrane Collaboration in a variety of different settings”* (page 18). The review also recommended the establishment of an External Advisory Board to which Cochrane would be accountable.^49^

In Gøtzsche and colleagues' definition of governance, nonscientists are expected to follow where scientific experts lead. The Governing Board's definition, in contrast, emphasizes accountability to external stakeholders as well as collaboration, consultation, communication, and conflict resolution. The former definition corresponds broadly to the structures and systems for overseeing the traditional, university‐led science that Gibbons et all called “Mode 1.” The latter corresponds to what is needed to support a more contemporary, co‐produced “Mode 2” kind of science.^50^

Gibbons et al describe Mode 1 science as *hegemonic* (that is, relating to domination) and driven by closed hierarchies of scientists and the institutions they lead.^50^ Mode 2 science, in contrast, is generated not only (or even primarily) in universities but also within its context of application—a heterogeneous and more or less democratic transaction space known as the “agora,” embracing state, economy, culture, and wider public sphere as well as academic institutions.

To be credible with its diverse audiences, suggest Gibbons and colleagues, Mode 2 knowledge must be seen as socially as well as scientifically robust (hence ethical, patient‐centred, environmentally sustainable, equitable, and a good use of public resources).^50^ In this kind of science, “… *the research process can no longer be characterised as an ‘objective’ investigation of the natural (or social) world, or as a cool and reductionist interrogation of arbitrarily defined ‘others’. Instead it has become a dialogic process, an intense (and perhaps endless) ‘conversation’ between research actors and research subjects”* (page 2).^51^

As other scholars have argued, Gibbons et al's Mode 1 and Mode 2 are not merely different approaches to how science is undertaken; they also embrace different perspectives on it should be governed—respectively, through hierarchies, rules and rational‐bureaucratic procedures or through collaboration, dialogue and other forms of dynamic engagement.^52^, ^53^

In a Mode 1 view of science, what is happening in the Cochrane Collaboration might justifiably be described as a failure of governance. But in Mode 2 science, the strategic shift from hegemonic governance to a more democratic, externally accountable, and dialogic form of governance is a robust and commendable approach. The acrimonious exchanges between Gøtzsche and the majority of the Governing Board can be understood philosophically as a confrontation between Mode 1 (in which power‐knowledge is held by academics) and Mode 2 (in which power‐knowledge is *generated by* a thriving multistakeholder network).

This resonates also with other influential approaches to understanding science in a changing society, such as the notion of postnormal science developed by Silvio Funtowicz and Jerome Ravetz in the early 1990s.^54^ These authors argue that in increasingly pluralistic and complex societies, science can no longer pretend to be an entirely value‐neutral enterprise insulated from the commitments and struggles of the societies in which it is embedded. Postnormal science needs to pay explicit attention to the management of uncertainty, to deal with a multiplicity of perspectives and commitments, and include a wide range of experts focused on solving concrete problems rather than attend exclusively to disciplinary or methodological purity.

The contrasting use of language and symbolic actions by different stakeholders in this debate illustrates a Foucauldian notion of power. Peter Digeser, extending the work of Stephen Lukes,^55^ proposes three progressively more critical questions about power in social situations: “Who, if anyone, is exercising power?”, “Whose issues have been mobilised off the agenda and by whom?”, and “Whose interests are being harmed?”.^56^ Drawing on Foucault, Digeser adds a fourth question: “What kind of subject is being produced?”

This fourth, Foucauldian, question—which pertains also to the types of relationships between actors that emerge through the exercise of power—radically reframes power: no longer is it something that one predefined individual or group of stakeholders wields over others but something that brings particular subjects (individuals, stakeholder groups) into being and enables them to have interests, desires, and choices (or not). The various social roles and relationships which we recognize in society—professor‐student, doctor‐patient, police officer‐criminal, and so on—are the result of historical and socio‐cultural practices that have given rise to, and legitimated, these relationships of power. In Foucault's words (cited in Digeser, page 981), *“power is co‐extensive with the social body; there are no spaces of primal liberty between the meshes of its network”*.

The crisis in Cochrane is not just a power struggle in the conventional sense but one that can be usefully analysed using the fourth (Foucauldian) face of power. What subjects, and what relationships, have been generated by key historical and socio‐cultural forces relevant to Cochrane's existence and activities? The emergence of evidence‐based medicine in the 1990s and 2000s, for example, created new kinds of experts and new kinds of expertise; prediction using average effect sizes derived from meta‐analyses of clinical trials came to overshadow the embodied wisdom of personal clinical experience as the basis of clinical decision‐making.^57^ Evidence‐based medicine (and the practices and forces that gave rise to it) also helped to generate the widely accepted assumption that policymaking is largely a matter of putting into practice insights from science and other types of systematic evidence (“what works?”), thus converting fundamentally political issues into scientific and technical ones.^58^, ^59^ Leaving aside questions raised by others about their own undeclared biases,^7^ Gøtzsche and his colleagues' considerable scientific authority, and their mandate to influence policy, is the product of these historical and cultural forces. In Foucauldian terms, the rise of evidence‐based medicine in a sense *created* Gøtzsche and experts like him.

But history, including the history of scientific thought, does not stand still. In recent years, evidence‐based medicine, and evidence‐based practice in other fields of social policy, has come to face its own epistemic crisis in which the assumption of the objective and dispassionate “view from nowhere” that can be unproblematically put into practice through a translational pipeline (broadly, Mode 1 science) has been questioned, both philosophically and practically.^38^ Concepts such as Gibbons et al's Mode 2 agora and Funtowicz and Ravetz' postnormal science described above call for a new, more democratic, and diverse set of power relations as well as a more diverse range of experts (including expert patients, expert policymakers, and expert commercial partners) and kinds of expertise.^50^, ^51^ Arguably, Gøtzsche et al's discursive efforts to present him as someone above scrutiny who can and should define the Cochrane brand are an attempt to resist the epistemic forces that have begun to redefine them as *a different kind of subject* (one that is considerably less powerful) and a relic of the past.^60^

## 4 RESOLVING THE CRISIS—OR HARNESSING THE CONFLICT PRODUCTIVELY?

This paper has argued that, notwithstanding awkward moments between particular personalities, recent events in Cochrane can be framed as an epic struggle for the organization's scientific, philosophical, and moral soul. To some extent, the struggle depicted in this paper reflects the wider crisis we have previously described within the evidence‐based medicine movement.^38^ Both illustrate the schism between a procedural and expert‐centred approach to best evidence and an alternative approach that is more “*socially distributed, application‐oriented, trans‐disciplinary and subject to multiple accountabilities”* (page 1).^51^

In the past 26 years, Cochrane's remit has expanded, and demand for its trusted outputs has risen. In parallel, roles have become formalized, work has differentiated, new hierarchies emerged, and governance structures evolved. To what extent has Cochrane abandoned its core principles and scientific standards to meet the expectations of those who fund and use its products? To what extent is its brand now tarnished?

As noted in our introduction, we do not seek to take a definitive position on the veracity of the different narratives discussed in this paper. We believe that if it is to make a difference, Cochrane's work must be externally accountable, engaged with key stakeholders and embedded in the wider health economy, but we also acknowledge that the methodology of systematic review is increasingly specialized and dependent on experts, and that commercial conflicts of interest can be subtle, insidious, and damaging in the review process.^35^, ^36^, ^37^, ^38^ In other words, we accept that whilst the narratives discussed above have been articulated in somewhat hostile language and are mutually incommensurable, they are also both to some extent “true.”

According to Chantal Mouffe's political philosophy, conflicts are at the centre of politics and are constitutive of society^61^: To politicize an issue is to represent the world in a conflictual manner *“with opposed camps with which people can identify”* (p 25).^62^ Drawing on Derrida's insistence on the irreducibility of difference,^63^ Mouffe claims that the production of identity and identification is at the same time the production of difference.^61^ To constitute a “we” always requires a “them.” By articulating divergent positions, we seek to provide the possibility for the formation of subjects or identities in relation to the issues in question. Such a public contestation shapes the social order—and also contains the seeds of its transformation.

Inspired by Mouffe,^61^ and also mindful that complex systems are characterized by tensions and paradoxes that cannot always be reconciled,^64^ we suggest that one way out of Cochrane's current crisis is to stop trying to resolve it. Counterintuitively, it may be more productive to accept that two incommensurable versions of the truth are likely to continue to coexist in permanent tension. Rather than asking “whose claim to the moral high ground is more legitimate, Gøtzsche's or Cochrane's?” (a once‐and‐for‐all question that has already generated considerably more heat than light), it may be useful to keep asking “how does the tension between Mode 1 governance (abstract scientific principles, uncompromisingly applied) and Mode 2 governance (pursuing scientific rigour along with policy engagement, fundraising, cross‐sector participation and relationship‐building, shared agenda‐setting and conflict resolution) play out in this particular situation?”

Articulating Cochrane's challenge in terms of an incommensurable tension between two philosophical perspectives allows us productively to harness the conflicts that gave rise to it, since both versions may provide insights when making complex judgements. Mouffe distinguishes between *antagonism*, which is a struggle between enemies in a war‐like situation, and *agonism*, which is defined as conflict between adversaries that share a commitment to addressing a problem through democratic means.^61^ But, she suggests, agonistic pluralism requires that the conflicts are acknowledged and openly discussed rather than hidden behind a veil of consensus. Such an approach may, perhaps, help protect against the dangers of toxic illusio referred to earlier where actors involved are spurred on by the game and fail to question the taken‐for‐granted processes and assumptions underpinning it.^23^

As noted, not all the facts of the Cochrane crisis are in the public domain. Challenging personalities and bitter interpersonal disputes are the kind of “soft intelligence” that is poorly captured by formal governance mechanisms.^65^ That acknowledged, we believe that the crisis in Cochrane is epistemic as well as individual and that whatever happens with Professor Gøtzsche, the Collaboration will continue to struggle with the underlying philosophical and political issues that his case has raised.

The current crisis has already inflicted deep wounds. They will be long to heal and forever etched in Cochrane's memory. Unless Cochrane and its supporters and critics can find a way of moving from an antagonistic (destructive) to an agonistic (constructive) approach to dealing with epistemic conflict, we fear that damaging clashes will be doomed to recur periodically between the Governing Board and the next generation of leaders of the evidence‐based medicine movement.

## CONFLICT OF INTEREST

TG is an author on several Cochrane reviews and protocols; she has peer‐reviewed Cochrane reviews in the past and has received second‐class travel fares and accommodation for speaking at Cochrane meetings.
